# Do Anti-Oxidants Vitamin D_3,_ Melatonin, and Alpha-Lipoic Acid Have Synergistic Effects with Temozolomide on Cultured Glioblastoma Cells?

**DOI:** 10.3390/medicines5020058

**Published:** 2018-06-20

**Authors:** Diane D. McConnell, Joe W. McGreevy, Macy N. Williams, N. Scott Litofsky

**Affiliations:** Division of Neurological Surgery, University of Missouri-Columbia School of Medicine, One Hospital Drive, MC 321, Columbia, MO 65212, USA; cundiffd@health.missouri.edu (D.D.M.); jwm3m8@health.missouri.edu (J.W.M.); macywilliams@mail.missouri.edu (M.N.W.)

**Keywords:** glioblastoma, antioxidant therapy, vitamin D_3_, melatonin, lipoic acid, Temozolomide, MTT, oxidative stress, neural stem cells

## Abstract

**Background:** Cancer patients often take over-the-counter anti-oxidants as primary treatment or in combination with chemotherapy. Data about such use in glioblastoma is limited. **Methods:** Cultured U87-MG cells, a primary glioblastoma cell line (MU1454), U87-MG derived stem-like cells (scU87), and MU1454 derived stem-like cell lines (scMU1454) were pre-treated with one of three anti-oxidants—Vitamin D_3_, Melatonin, and alpha-lipoic acid (LA)—for 72 h, followed by a 72 h treatment with temozolomide (TMZ). MTT (3-(4,5-Dimethylthiazol-2-yl)-2,5-diphenyltetrazolium bromide) assessed cell proliferation. DCFDA Cellular ROS Detection Assay and Glutathione peroxidase (GP×1) activity assessed the anti-oxidant effect of TMZ +/− an anti-oxidant drug. **Results**: Vitamin D_3_ did not affect MU1454, but had slight TMZ synergism for U87-MG. Melatonin 1 mM decreased U87-MG and MU1454 cell proliferation. As pretreatment to TMZ, melatonin 1 mM and 50 nM significantly reduced proliferation. LA 1 mM had a significant effect alone or with TMZ on U87-MG and MU1454. LA 250 uM also reduced proliferation by almost 50%. Melatonin and LA significantly enhanced the responsiveness of scMU1454 to TMZ, while Melatonin 50 nM exerted similar effects on scU87. The anti-oxidants were associated with generally decreased reactive oxygen species and limited GP×1 effects.** Conclusions:** Anti-oxidants may have synergistic effects with TMZ. LA offers the most promise, followed by melatonin.

## 1. Introduction

Glioblastoma multiform (GBM) has an aggressive growth pattern, leading to poor prognosis despite maximal surgical resection followed by combined adjuvant radiation and chemotherapy. The two year overall survival rate for newly diagnosed GBM patients in the late 1980’s–90’s was 3.3%, increasing to about 20–48% more recently [1,2,3,4,5,6]. Temozolomide (TMZ), an oral imidazotetrazine second-generation alkylating agent, has contributed greatly to improving the overall survival in GBM patients since its introduction to the arsenal in the early 2000′s; it is now a mainstay of the current standard treatment regimen [3,7]. One of the unique features of TMZ is that it enters the cerebrospinal fluid [7]. Standard dosing regimen for the treatment of GBM is an initial concomitant phase in which TMZ is given once daily with conformal radiotherapy for 42 days, followed by a maintenance phase in which TMZ is given 150 mg/m² by mouth (PO) every day for five days, followed by a “rest period” of 23 days. This maintenance phase is repeated at 28-day cycles for a total of six or more cycles [8]. Some GBM are resistant to treatment with TMZ, especially those with hypomethylation of the O^6^-methylguanine methyltransferase (MGMT) promoter [9]. Given the lack of complete satisfaction of the current standard medical treatment, the strategy of co-treating brain tumors with supplemental over-the-counter (OTC) therapies is an option that offers hope to many brain cancer patients [10].

In recent years, potential chemotherapeutic properties of anti-oxidants have been examined as either a primary agent or in combination with an already established chemotherapeutic agent for different types of cancers. Controversy has been raised among practitioners about the efficacy and safety of these complimentary treatments and their potential role in protecting tumor cells from conventional therapy [11]. In GBM, a high metabolic rate and increased basal levels of reactive oxygen species (ROS) play an important role as chemical mediators in the regulation of signal transduction, cellular proliferation, and protecting malignant cells from apoptosis [12]. In addition, studies have shown that a higher level of ROS in cancer cells of all types leads to increased stress on DNA repair mechanisms, as well as disrupting membranes and protein stability [13]. Considering the role that ROS plays in tumorigenesis, over-the-counter anti-oxidants may present themselves as a novel anti-cancer therapeutic modality. Reducing high levels of ROS by scavenging free radicals could allow the stabilization of a wide range of cellular mechanisms. Anti-oxidants may also have a compounding effect by stimulating the activity of antioxidant enzymes, such as glutathione peroxidase (GP×1), further decreasing the level of ROS inside the cell [14].

Several naturally occurring anti-oxidants of interest possess the ability to cross the blood-brain-barrier, including Vitamin D_3_, Melatonin, and Lipoic Acid. These three supplements have been shown in clinical and experimental studies to enhance the cytotoxic efficacy of chemotherapy for some tumors [15]. Other potential mechanisms could also be targeted by these agents. For instance, a small subset of cancer cells known as brain tumor stem-like cells implicated in the neoplastic transformation to GBM are also thought to contribute to treatment failure by the escape of these stem cells following conventional therapy such as TMZ [16]. Both Vitamin D_3_ and melatonin have been implicated in playing a role in altering these stem-like cells and therefore inhibiting GBM cell proliferation [16,17].

Previous studies investigating the combined treatment of dietary supplements and TMZ have shown that the combination of TMZ with natural products may be more effective than TMZ alone [17,18]. The aim of this pilot study was to examine the supplemental influence of Vitamin D_3_, melatonin, and lipoic acid anti-oxidants on TMZ anti-cancer activity in cultured GBM cells measuring changes in proliferation via the MTT Cell Proliferation Assay. We hypothesized that anti-oxidants used in combination with TMZ would enhance TMZ’s anti-proliferative effects. We examined the effects of pre-treatment of the cells with the anti-oxidant (i.e., mimicking the patient use of these drugs during the “rest period”) to determine if they have the ability to sensitize the remaining tumor cells to TMZ. Because anti-oxidants are thought to modulate enzymatic regulation and influence the production of reactive oxygen species, we assessed these mechanisms as well [11,14].

## 2. Materials and Methods

### 2.1. Reagents

The reagents used in the experiments were purchased as follows: fetal calf serum (FCS) (16010159) (Invitrogen/Thermofisher, Waltham, MA, USA; TrypLE™ Express (12604-013), Pen Strep (15140-122), DMEM (1x) (31053-028), and DMEM/F-12 (11039-021) from GibcoLife Technologies, Grand Island, NY, USA; F-12K (30-2004) from ATCC, Manassas, VA, USA; dimethyl sulfoxide (DMSO) (D-2650) and Vybrant^®^ MTT Assay (V13154), (R)-(+)-α-Lipoic acid (07039-10 mg), 1a,25-DiHydroxyvitamin D_3_ (D1530), and Melatonin Crystalline (M5250) from Sigma-Aldrich, St Louis, MO, USA.

A stock solution of each anti-oxidant was prepared by dissolving them in 95% EtOH. For cellular treatment, stock solutions were dissolved in the appropriate culture media for the respective cell line. Final concentrations of EtOH did not exceed 0.1% (*v*/*v*). A stock solution of TMZ was prepared by dissolving the sample in DMSO, with treatment solution prepared by dilution in the appropriate culture media. Final concentration of DMSO did not exceed 0.1% (*v*/*v*).

### 2.2. Cell Culture and Treatments

Two GBM cell lines were used for this study. The U87-MG (human GBM) cell line was obtained from the American Tissue Culture Collection (HTB-14™) (ATCC, Rockville, MD, USA). These cells were seeded in two BioLite vented 75 cm flasks (cat no. 130190, ThermoFisher, Rochester, NY, USA) in Dulbecco Modified Eagle Medium supplemented with 10% Fetal Bovine Serum and 1% penicillin-streptomycin in a humidified incubator with 5% CO_2_ at 37 °C. Human GBM primary cell line (MU1454) was harvested from a surgically resected, pathologically confirmed specimen of a patient of Dr. Litofsky, Division of Neurological Surgery, University of Missouri, Columbia, MO, USA. This primary cell culture was established following the ethical procedures as outlined in the IRB protocol approved by the University of Missouri Institutional Review Board (#1044138). MU1454 cells were cultured with Dulbecco Modified Eagle Medium/F-12 containing no Phenol Red, Fetal Bovine Serum (20%) and Penicillin-Streptomycin (1%) in a humidified incubator with 5% CO_2_ at 37 °C and were passaged by TrypLE™ every three to four days at confluence.

The U87-MG neural stem-like cell (scU87) culture was isolated from the growing U87-MG cell line. Monolayer cell lines were seeded at a density of 100,000 cells/mL in laminin (L-2020, Sigma, Tokyo, Japan) coated flasks in defined serum-free medium, DMEM/F12, supplemented with 1% Penicillin/Streptomycin, Epidermal Growth Factor (Invitrogen) (20 ng/mL), Basic Fibroblast Growth Factor (Sigma) 20 ng/mL, B-27 supplement (50×) without Vitamin A (Gibco), 2 mL/100 mL, N-2 Supplement (100×) (Invitrogen) 1 mL/100 mL, Insulin (Sigma I1882, stock 25 ug/uL) 80 uL/100 mL, and BSA (Sigma, A8412, 7.5%) 100 uL/100 mL. Alternately, Neuroblast™ plus Medium with B-27™ Plus Supplement (50×) was used for maintenance of the cells (GIBCO, A3582901). The MU1454 stem-like cell (scMU1454) culture was started in the same media at the time of surgical resection.

The following primary antibodies were used on cytospin preparations to confirm the cells’ positivity as brain tumor-initiating cells: CD133 1:2000 (MA5-15875, Pierce, Rockford, IL, USA), Nestin 1:400 (Ma1-46100, Pierce, Rockford, IL, USA), Sox2 1:100 (hSOX2, R&D Systems, Minneapolis, MN, USA), and neuron-specific class III beta-tubulin 1:100 (PA1-41331, Pierce, Rockford, IL, USA). Briefly, cells were spun at 700 rpm for 5 min onto positive charged slides using a Cytopro 7620 cytocentrifuge. Slides were air-dried, fixed in formalin, subjected to antigen retrieval, incubated with the primary antibody, and detected using the Vector Impress Peroxidase Polymer system and DAB chromogen.

### 2.3. MTT Cytotoxicity Assay

Initially, the effect of the antioxidants on the viability of U87-MG and MU1454 were studied after 24 h, 48 h, and 72 h of treatment to determine an effective dosing schedule for the anti-oxidants. Later studies focused on pre-treatment at 72 h when it was determined that the maximum effects could be attained at this end point. Cells were plated when the flask confluency reached 95% or greater. After determining the cell count with a hemocytometer, cells were plated at a density of 3 × 10^3^ cells per well into a Falcon 96-multiwell plate and left to incubate for 24 h (Cat no. 353072, Becton Dickinson Labware, Franklin Lakes, NJ, USA). Cells were treated and cultured using Vitamin D_3_ at concentrations of 1 nM, 10 nM, and 100 nM, Lipoic Acid at concentrations of 100 nM, 250 µM, and 1 mM, and Melatonin at concentrations of 1 mM, 1 µM, and 50 nM. Dosages were determined from published LC_50_ values and working dosages in the literature that could approximate natural physiologic levels if taken orally [19,20,21,22,23,24].

Cell proliferation was evaluated by the MTT assay, as previously described [25]. Briefly, the cells were cultured in 96-well plates and incubated with 10 µL of MTT (12 mM) in a CO_2_ incubator for 4 h. A total of 100 µL of SDS-HCl solution was added to each well and returned to the CO_2_ incubator for 4 h, at which time the optical density at 570 nm was measured using a microplate reader (ELx-800, BioTek, Winooski, VT, USA). MTT assays were performed on a total of fifteen wells per treatment(s) per cell line and the experiment was repeated in triplicate.

In order to determine if the previous drugs have an antagonistic or synergistic effect with Temozolomide, a second study was performed. Cells were plated as described above and at 24 h, the cultures were treated with Melatonin, Lipoic, or Vitamin D_3_ at the dosages described above. The cells were incubated for 72 h at standard conditions (5% CO_2_ at 37 °C). The media was removed after 72 h and replaced with fresh media containing TMZ 50 µM or 100 µM (S1237, Selleckchem, Houston, TX, USA). The plates were returned to the incubator for 72 h. At each endpoint of treatment, cell proliferation was evaluated by the MTT assay as described earlier [25]. Control wells included treatment with anti-oxidant only, TMZ only, and a negative control containing only media. MTT assays were performed on a total of nine wells per treatment(s) per cell line and repeated in triplicate. Additional experiments were repeated using TMZ concurrently with the antioxidants for 72 h following pre-treatment for 72 h.

### 2.4. Measurement of ROS Production

Reactive oxidative species (ROS) production was measured by using the DCFDA Cellular ROS Detection Assay Kit from AbCam (ab113851). Briefly, cells were harvested and seeded at 2.5 × 10^4^ cells per well on a black walled, fluorescent 96-well plate. Cells were allowed to attach overnight. Cells were stained with DCFDA 25 µM in 1× buffer and incubated for 45 min at 37 °C. After washing with 1× buffer, the cells were treated with 100 µL/well of varying concentrations of anti-oxidant +/− TMZ. Measurement of absorbance/ROS was made at multiple time points: 20 min, 1 h, 1.5 h, 2, 3, 4, 5, 6, and 7 h at Ex/Em: 485/535 (485/529) on a BioTek Synergy 4 multi-detection plate reader. The data is reported as the percentage of control after removing the background signal.

### 2.5. Glutathione Peroxidase Activity Assay

Glutathione Peroxidase activity (GP×1) was determined using the Glutathione Peroxidase Assay kit from Cayman Chemicals (#703102). Briefly, following treatment, cells were harvested using a rubber policeman and homogenized using a mortar and pestle in cold buffer, centrifuged at 10,000 g for 15 min, and the supernatant was collected. Cell extracts were normalized to a protein concentration using the BCA assay. Afterwards, samples (20 µL) were incubated in assay buffer (Tris-EDTA) (100 µL) and co-substrate mixture which contained NADPH, glutathione, and glutathione reductase (50 µL). Assays were performed in triplicate for each sample. The reaction was initiated by adding cumene hydroperoxide (20 µL). After mixing for a few seconds, the reaction was read at 340 nm using a BioTek ELx800 plate reader every minute for five time points. Change in absorbance was calculated and used to determine the GP×1 (nmol/min/mL).

### 2.6. Statistical Analysis

MTT values were reported as the mean percentage of controls ± SD. All other data values were reported as mean ± SD. Differences between groups were tested with a student t test with significant differences determined at *p* values <0.05. Figures were generated in GraphPad Prism with Error bars representing a 95% confidence interval.

## 3. Results

### 3.1. Vitamin D_3_ Effect on Cell Proliferation

**Figure 1:**
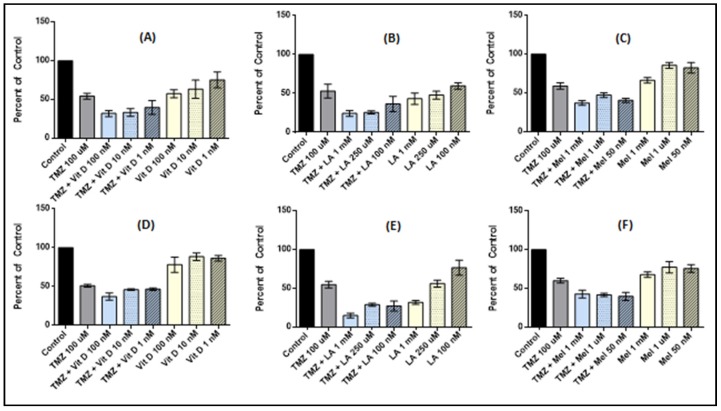
Cell proliferation of U87-MG (**A**–**C**) and MU1454 (**D**–**F**) measured via the MTT assay after serial (“cycled”) 72 h treatments with antioxidants (Vitamin D [VitD], Lipoic Acid [LA], or Melatonin [Mel]) followed by Temozolomide (TMZ) at 100 uM. Dose of TMZ is constant at 100 uM, while dose of antioxidant varies and is indicated on the graph. Error bars represent the 95% confidence interval.

Vitamin D_3_ was tested in both U87-MG and MU1454 cells lines for its anti-proliferative activity as both an individual agent and as a pre-treatment with TMZ 100 µM (Figure 1A,D). Generally, U87-MG appears to be more sensitive to treatment with vitamin D_3_ when compared to MU1454. In the U87-MG cells, individual treatment with vitamin D_3_ resulted in a step-wise dose response as the vitamin D_3_ dose was increased. This response reaches its peak at 100 nM with activity at about 60% that of the control (*p* < 0.0001). When used as a pre-treatment in combination with TMZ, vitamin D_3_ showed moderate synergistic activity. However, this action peaks at the 100 nM and 10 nM groups at 70% that of the TMZ control (*p* < 0.0001). No differences between the 10 nM and 100 nM groups were present (*p* = 0.2280).

MU1454 individual treatment with vitamin D_3_ provided a minimal anti-proliferative effect. At 100 nM, despite the effect being minimal, it did meet significance (*p* = 0.0443). When used as a pre-treatment in combination with TMZ, vitamin D_3_ also showed minimal synergistic activity. However, the highest dose group (100 nM) showed activity at about 80% that of the TMZ only group (*p* < 0.0001).

**Figure 2:**
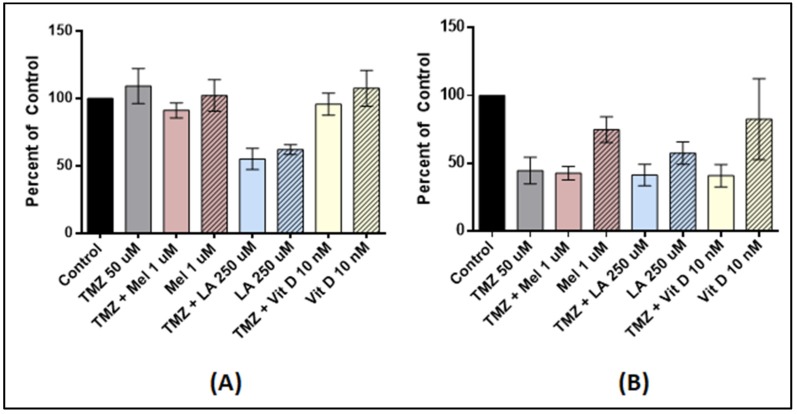
Cell proliferation of U87-MG and MU1454 measured via the MTT assay after serial (“cycled”) 72 h treatments with antioxidants (Vitamin D [Vit D], Lipoic Acid [LA], or Melatonin [Mel]) followed by TMZ 50 uM. Dose of TMZ is constant and the doses of antioxidants are indicated on the graph. (**A**) U87-MG cells after treatment with Melatonin at 1 uM, Lipoic acid at 250 uM, and Vitamin D at 10 nM with or without TMZ at 50 uM. (**B**) MU1454 proliferation after treatment with melatonin at 1 uM, Lipoic acid at 250 uM, and Vitamin D at 10 nM with or without TMZ at 50 uM. Error bars represent the 95% confidence interval.

Since lower doses of chemotherapy generally result in less toxicity, we also tested the use of TMZ 50 µM and Vitamin D_3_. In U87-MG, TMZ 50 µM cycled with Vitamin D_3_ for 24 h (*p* = 0.9218), 48 h (*p* = 0.1088), and 72 h (*p* = 0.0614) had no effect (data only shown for 72 h). TMZ 50 µM was effective in reducing the proliferation of MU1454 by 52% (*p* = 0.0002), but no significant synergistic effect was afforded by the pre-treatment with Vitamin D_3_ (*p* = 0.5031) (Figure 2).

### 3.2. Lipoic Acid Effects on Cell Proliferation

Lipoic acid 1 mM had the most significant effect both individually and in sequence with TMZ 100 µM on both U87-MG and MU1454 (*p* < 0.0001). When treated at concentrations attainable physiologically (250 µM), a significant reduction in cell proliferation also occurred, with cell proliferation at almost 50% that of the controls and TMZ controls in both cell lines (U87-MG and MU1454 (*p* < 0.0001) (Figure 1).

TMZ 50 µM preceded by incubation with lipoic acid at 250 µM effectively reduced the proliferation of U87-MG by 44% at 72 h (*p* < 0.0001). Little to no synergistic action with TMZ occurred at this concentration on the MU1454 (*p* = 0.5385) (Figure 2).

### 3.3. Melatonin Effects on Cell Proliferation

Melatonin most effectively decreased cell proliferation as a solo agent at 1 mM for both cell lines (*p* < 0.0001). As a pretreatment to TMZ 100 µM, both Melatonin 1 mM and 50 nM successfully reduced cellular proliferation in MU1454 by 60% and 65%, respectively, compared to the controls (*p* < 0.0001). The same concentrations in U87-MG reduced proliferation by 60% and 55% compared to the controls (*p* < 0.0001). The same doses reduced proliferation by about 40% when compared to TMZ alone (*p* < 0.0001) (Figure 1).

In U87-MG, Melatonin 1 µM followed by TMZ 50 µM resulted in no change in proliferation at 24h (*p* = 0.8542) and 48h (*p* = 0.4830), and only had a modest effect at 72 h (9% reduction) compared to TMZ control cells (data only shown for 72 h; *p* = 0.0099). TMZ 50 µM following Melatonin 1 µM pre-treatment did not demonstrate any synergistic effect for MU1454 (*p* = 0.6659) (Figure 2).

### 3.4. Effect of Anti-Oxidant and TMZ Co-Treatment on Proliferation

**Figure 3:**
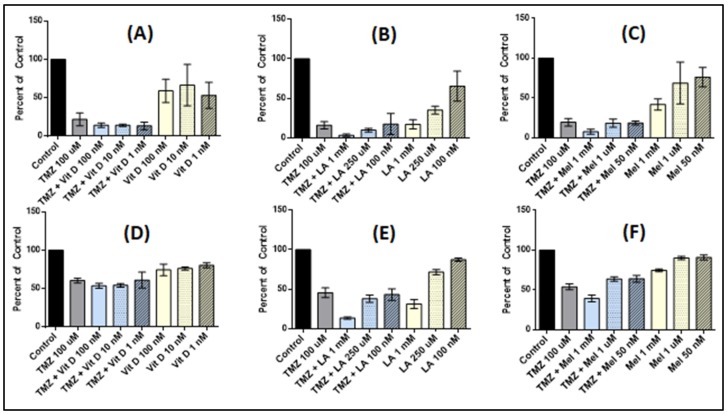
Cell proliferation of U87-MG and MU1454 measured via the MTT assay after serial 72 h treatments with antioxidants (Vitamin D [Vit D], Lipoic Acid [LA], or Melatonin [Mel]), followed by combined 72 h treatment with antioxidants and TMZ at 100 uM. Dose of TMZ is constant at 100 uM, while dose of antioxidant varies and is indicated on the graph. (**A**) U87-MG cells after treatment with Vitamin D at 1 nM, 10 nM, and 100 nM. (**B**) U87-MG cells after treatment with Lipoic acid at 1 mM, 250 uM, and 100 nM. (**C**) U87-MG cells after treatment with Melatonin at 1 mM, 1 uM, and 50 nM. (**D**) MU1454 cells after treatment with Vitamin D at 1 nM, 10 nM, and 100 nM. (**E**) MU1454 cells after treatment with Lipoic Acid at 1 mM, 250 uM, and 100 nM. (**F**) MU1454 cells after treatment with melatonin at 1 mM, 1 uM, and 50 nM. Error bars represent the 95% confidence interval.

As these anti-oxidants are available over-the-counter, making them easily accessible for patients, we tested whether or not co-treatment of antioxidants with TMZ 100 µM after anti-oxidant pre-treatment affects the cellular proliferation of U87-MG or MU1454 (Figure 3). In both U87-MG and MU1454 cells, the only significant decrease in cellular proliferation with co-treatment was observed in the TMZ + Melatonin 1 mM and TMZ + Lipoic acid 1 mM groups when compared to TMZ only (*p* < 0.001). Other groups (such as vitamin D_3_ and lipoic acid 250 uM) demonstrated insignificant diminution in proliferation (*p* > 0.05).

### 3.5. Antioxidant Pre-Treatment on Tumor Stem-Like Cells

**Figure 4:**
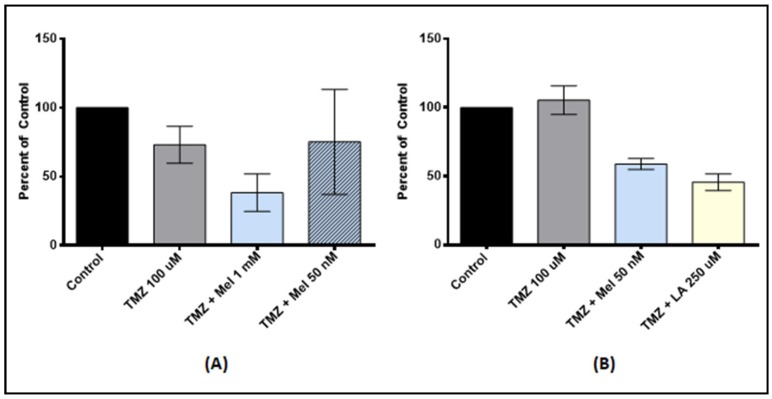
Cell proliferation of U87-MG stem-like cells (**A**) and MU1454 stem-like cells (**B**) measured via the MTT assay after serial 72 h treatments with antioxidants (Lipoic Acid [LA] or Melatonin [Mel]) followed by TMZ at 100 uM. Dose of TMZ is constant at 100 uM, while dose of antioxidant varies and is indicated on the graph. (**A**) scU87 cells after pre-treatment with melatonin at 1 mM and 50 nM followed by TMZ. (**B**) scMU1454 cells after pre-treatment with melatonin at 50 nM and Lipoic acid at 250 uM followed by TMZ. Error bars represent the 95% confidence interval.

Because cancer stem-cells may play a role in allowing cancer to escape chemotherapeutic treatments, we wanted to test whether anti-oxidant pre-treatment enhanced the action of TMZ on tumor stem-like cells (Figure 4). We used melatonin and lipoic acid because these anti-oxidants had the most robust synergistic effect in U87-MG and MU1454. In scU87, pre-treatment with melatonin 1 mM followed by TMZ 100 µM offered a significant reduction in proliferation compared to the TMZ control (*p* = 0.0011). scU87 displayed no significant synergism with pre-treatment at 50 nM (*p* = 0.9228). Melatonin 1 mM pre-treatment was also effective in the scMU1454 cell line, with a 45% reduction (*p* < 0.0001). In addition, scMU1454 pre-treated with lipoic acid 250 µM followed by TMZ 100 µM had a synergistic reduction of proliferation by nearly 55% (*p* < 0.0001).

### 3.6. ROS Production

**Figure 5:**
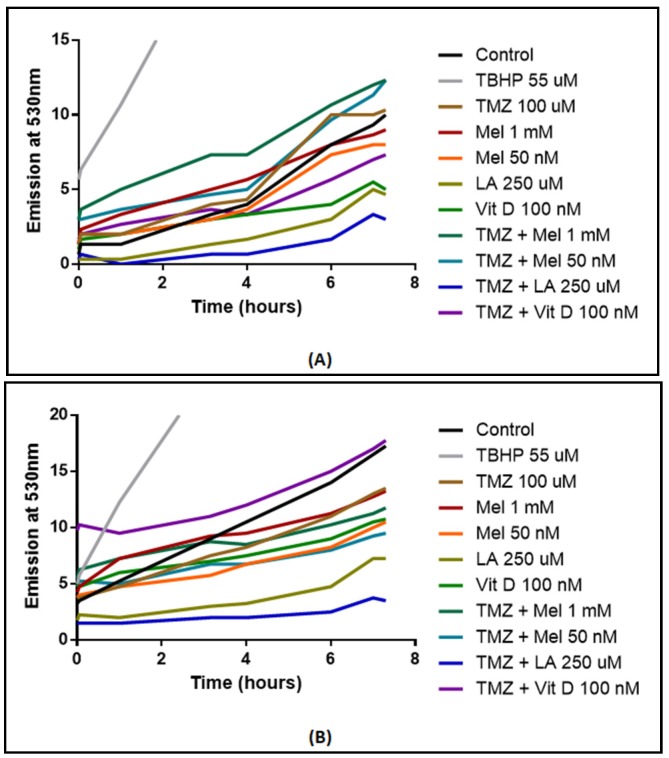
Reactive Oxygen Species (ROS) measured via 2′,7′-dichlorofluorescin diacetate (DCFDA) fluorophore in U87-MG (**A**) and MU1454 (**B**) after treatment with antioxidants (Vitamin D [Vit D], Lipoic acid [LA], or Melatonin [Mel]) and/or temozolomide (TMZ) at 100 uM. Dosages of antioxidants are specified on the graph. Positive control was 55 μM of tert-butylhydroperoxide (TBHP).

ROS production was measured in U87-MG and MU1454 after either solo anti-oxidant treatment or co-treatment with TMZ 100 µM and anti-oxidants (Figure 5). In U87-MG, co-treatment with anti-oxidants and TMZ generally decreased ROS production when compared to TMZ alone, with the most robust response observed with co-treatment of lipoic acid 250 µM and TMZ (*p* = 0.0072). In MU1454 cells, a similar response was observed (*p* < 0.0001). On the other hand, co-treatment with vitamin D 100 nM and TMZ had a similar ROS production as the controls (*p* = 0.7811), though it was significantly above that of the TMZ only treatment (*p* = 0.0234).

### 3.7. Glutathione Peroxidase Activity

**Figure 6:**
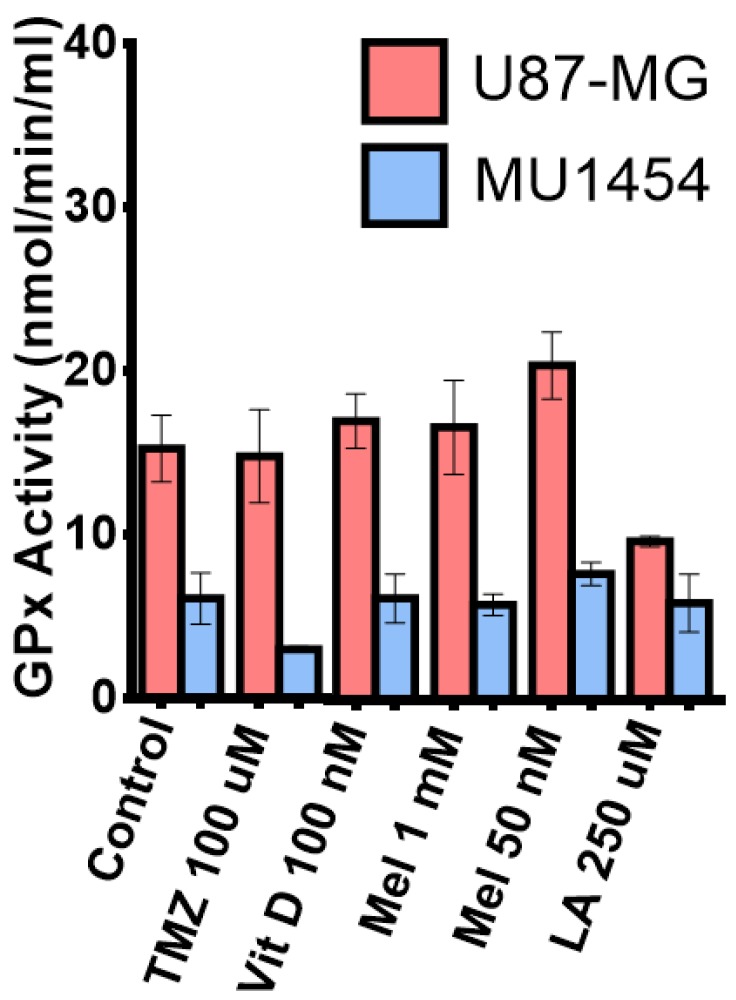
Measurement of Glutathione Peroxidase 1 (GP×1) activity in U87-MG and MU1454 after treatment with antioxidants (Vitamin D [Vit D], Lipoic acid [LA], or melatonin [Mel]) or Temozolomide (TMZ).

We measured the effect of individual antioxidant and TMZ 100 µM treatment on glutathione peroxidase activity (GP×1) in U87-MG and MU1454 to assess the antioxidant effects on the cellular ROS “buffering” system (Figure 6). In U87-MG, treatment with melatonin 50 nM increased GP×1 activity, while Melatonin 1 mM only had a minimal effect (50 nM: *p* = 0.0359; 1 mM: *p* = 0.5250). Surprisingly, treatment with lipoic acid 250 µM resulted in a robust decrease in GP×1 activity in U87-MG (*p* = 0.0096). Generally, MU1454 GP×1 activity was much less than U87-MG (*p* < 0.05) and that cell line also had a differential response to treatment. In MU1454, treatment with TMZ 100 µM displayed a robust decrease in GP×1 activity (*p* < 0.0263). Melatonin 50 nM treatment in MU1454 resulted in a slight, but not significant, increase in GP×1 activity (*p* = 0.1804); however, this increase was not as pronounced as that seen in U87-MG. In combination with TMZ, Vit D_3_ treatment in U87 was significantly decreased (*p* = 0069), while conversely showing a significant increased GP×1 activity in MU1454. TMZ + Mel 50 µM and TMZ + LA combination treatment resulted in a very significant increased GP×1 activity in U87-MG (*p* = 0081 and *p* = 0.0043 respectively), but showed no effect on the MU1454 cell line (*p* = 0.9664 and *p* = 0.1821). The combination of Melatonin 1 mM with TMZ was very similar to what was seen with Melatonin 1 mM alone, showing no effect on decreasing GP×1 activity on either cell line (U87 *p* = 0.1877 and MU1454 *p* = 0.6370).

## 4. Discussion

In this small pilot study, we evaluated the effect of three different anti-oxidant supplements alone and in combination with TMZ on the proliferation of two GBM lines and two stem-like cell lines. We examined the three anti-oxidant supplements because two of the supplements—Vitamin D_3_ and melatonin—have recently received much attention as an adjuvant therapy along with chemotherapeutics against GBM and other cancers. LA was of interest because recent work suggested LA might protect cells from the effect of chemotherapeutic agents [24]. These supplementations are widely available by an oral route and can cross the blood-brain barrier to achieve therapeutic levels.

We showed that each anti-oxidant decreased the proliferation of U87-MG and MU1454. Vitamin D_3_ had the least dramatic effect, especially for MU1454. Others have reported similar findings in GBM cell lines (Tx3095, Tx3868, U87, U118, U373). However, some cell lines, such as the rat glioma cell line, have responded favorably to Vitamin D_3_ in vitro [26]. Depending on the cell environment, media growth factors, and different molecular profiles, such as the presence or absence of Vitamin D receptors (VDSR), Vitamin D_3_ can have either proliferative or anti-proliferative effects [27]. Our doses of Vitamin D_3_—1 nM, 10 nM, and 100 nM—might have been too low to have an effect. On the other hand, higher concentrations are not physiologically attainable in human serum, and even less attainable in the brain. For example, Magrassi, et al. [28] successfully induced a significant reduction (>50%) of growth in two glioma cell lines (HU70 and HU197), but required doses over 5 µM. Furthermore, blood levels above or equal to 375 nmol/L or 150 ng/ML are considered toxic, resulting in hypervitaminosis D which causes hypercalcemia, renal stones, and bone demineralization, among other adverse effects [29].

In contrast, melatonin had a robust anti-proliferative response in both our cell lines—both when used as an individual agent and as pre-treatment to TMZ—consistent with other studies demonstrating the beneficial effects of melatonin combined with other chemotherapeutics and in other cancer types [30,31]. One concern, however, is that the commonly used concentration of melatonin (1 mM) may not actually be physiologically attainable [32,33]. Therefore, we included three different concentrations to address this issue. We observed a synergistic effect from melatonin 1 mM, as well as at the lower concentration of 50 nM. The 50 nM dose closely mimics physiological levels (54 nM) for patients taking melatonin 20 mg orally [34]. Interestingly, melatonin 1 µM did not exert any significant effect, replicating literature reports of antioxidants’ variability of actions [35]. Anti-oxidants, melatonin included, may directly scavenge free radicals in high (mM) concentrations. Conversely, at low (nM) concentrations, melatonin can act through receptors to increase the activity and expression of enzymes. [36,37,38] Hence, we see increased GP×1 activity at 50 nM, but not at 1 mM.

LA showed the most promising results in this study. Significant anti-proliferative activity was present as an individual agent and as pre-treatment to both U87-MG and MU1454. With low dose TMZ, tumor stem-like cells of both cell lines were sensitive to LA effects, which was robust even at a physiologic dose of 250 µM. Interestingly, LA has been shown to cause hypermethylation of the MGMT promoter, resulting in decreased MGMT proteins in glioblastoma [39]. Since hypomethylation of MGMT confers the resistance of glioblastoma to TMZ, our data provides a logical rationale for the co-treatment of TMZ with lipoic acid [40].

In this study, we were primarily interested in the tumor cell response to the pre-treatment of the cells with an anti-oxidant and its ability in potentiating the effects of TMZ on these cells. When considering the advantage of using complementary supplements in conjunction with chemotherapy, it has been suggested that the agent in question may help decrease the dosage of the chemotherapeutic drug by enhancing the cyotoxic effects of the anticancer agent, without losing oncological actions [11]. Toxicity and adverse side effects of chemotherapeutic drugs impact the quality of life of cancer patients and are often the reason why patients discontinue the treatment [41]. The effect of the antioxidants on healthy tissues is beyond the scope of this in vitro study; however, we tested two doses of TMZ to determine if there was a synergistic response that could suggest that a lower dose of TMZ could be used. The synergy of LA 250 µM with TMZ 50 µM on U87-MG supports this strategy; however, one must be cautious as effects can be different between different tumors, as we have shown in our small sample.

Another advantage, beyond the scope of this study, includes the protection of healthy cells, which also would help decrease toxicity. A key point to consider in the use of over-the-counter medications, and anti-oxidants in particular, is that the agent should not interfere with the cytotoxic effects of prescribed chemotherapeutic agents. Our in vitro results show only anti-oxidant augmentation or no effect on TMZ. No inhibition of TMZ effect by any of the anti-oxidants was observed, supporting the patient practice of supplementing their care with this type of alternative therapy.

We attempted to elucidate the mechanisms by which the three anti-oxidants exerted their effects on these parental cell lines by measuring ROS and GP×1 levels. ROS, including the superoxide anion (O_2_–), hydrogen peroxide (H_2_O_2_), and hydroxyl radical (HO), act as regulators or secondary messengers of signal transduction pathways for cell proliferation, survival, and apoptosis [42,43]. If ROS is present in very high, but non-lethal concentrations in cells, the redox balance shifts and results in cellular adaptations. Tumor cells use these adaptions to promote survival, proliferation, angiogenesis, and metastasis [43]. In fact, tumor cells have increased ROS generation compared to their non-cancerous counterparts [44].

While TMZ functions primarily as an alkylating agent affecting the methylation of DNA, TMZ also increases ROS production in gliomas [40]. Anti-oxidants can exert effects by either scavenging free radicals, or conversely, increasing intracellular ROS to lethal levels, causing cell death. They can also provide lasting effects by altering enzyme levels within the cell. For example, glutathione peroxidase uses glutathione (GSH) to deactivate ROS inside the cell [13,45,46,47]. TMZ resistance in glioma cell lines has been linked to a more robust ROS buffering system inside the cell. In one study, baseline GP×1 activity was directly associated with TMZ resistance, where higher GP×1 activity was found to increase TMZ resistance [47,48]. Higher GP×1 activity (and similarly higher GSH) results in stabilization of the redox environment, protecting the cell from apoptosis [47,48].

Both MU1454 cells and the U87-MG cells were sensitive to the TMZ concentrations used in this study; therefore, baseline ROS and GP×1 levels of the control cells did not confer resistance. While we did observe Vitamin D_3_-related change in ROS production in MU1454 when used as a solo agent, GP×1 activity was no different from the controls. In combination with TMZ, Vit D_3_ had no significant effect. These results support the impression that Vitamin D_3_ alone can act as a pro-oxidant by working with TMZ to increase the generation of ROS in tumor cells [49]. Koren, et al. [49] demonstrated that Vitamin D_3_ alone can exert its effect by increasing the ROS concentration in the absence of changes in GP×1 activity or glutathione levels in the cell. Table 1 illustrates the compilation of these changes.

Melatonin at high concentrations (1 mM) acts as a scavenger of free radicals (lowering ROS) and at lower concentrations (Nano molar concentrations) increases GP×1 activity [14]. In our studies, both the high concentration (1 mM) and lower concentration (50 nM) augmented the effects of TMZ on cellular proliferation, but not at the middle concentration of 1 µM. Both 1 mM and 50 nM concentrations exhibited similar activity for ROS production when paired with TMZ. However, in U87-MG cells, it increased ROS production over the control and TMZ-control cells (demonstrating a synergistic response), whereas MU1454 total ROS production with TMZ was less than the control and TMZ-treated controls, conversely suggesting that scavenging occurred. Despite increased GP×1 activity with the lower concentration alone or in combination with TMZ, we do not see a significant difference between the two concentrations in proliferation of the cell lines to conclude that this GP×1 activity played a role in explaining the mechanism of action for melatonin at the lower concentration. The significantly higher GP×1 activity also did not appear to confer resistance to TMZ in our cell lines, as suggested in the literature [47,48].

LA has concentration-dependent pro-oxidant and anti-oxidant properties [50]. LA significantly increases glutathione levels in the liver, lung, and kidneys of laboratory animals and can function as an ROS scavenger and stimulate or inhibit the activity of anti-oxidant enzymes [43,50,51]. Lipoic acid 250 uM significantly lowered ROS production in both cell lines, especially in combination with TMZ, as well as significantly lowered GP×1 activity. LA’s favorable response of inhibiting cell proliferation may be due to its ability to decrease the ROS burden of the cell as a free radical scavenger. Paradoxically, LA also significantly lowered GP×1 activity for U87-MG, but not for MU1454, which mimics that of the control and TMZ controls. Of special note is the significant increase in GP×1 activity when LA is used in combination with TMZ for U87-MG.These conflicting results suggest that the effect of LA on decreased proliferation of the two cell lines may well be through a ROS-independent cell death. Even though LA plays an active role in silencing MGMT, U87-MG is typically thought to not have MGMT hypomethylation, so an ROS mediated action is plausible [52].

Treatment failure for GBM has been attributed to the escape of stem-like tumor cells following conventional therapy [53]. Both Vitamin D_3_ and melatonin have been implicated in playing a role in altering these stem cells and therefore inhibiting GBM proliferation [54,55]. Our study showed that scMU1454 was non-responsive to TMZ, supporting the possible resistance of these cells to this agent Additionally, we were able to demonstrate that these same cells were significantly affected by Melatonin and LA when followed by TMZ treatment. scU87-MG was sensitive to TMZ alone, and demonstrated a statistically significant response to Melatonin 1 mM followed by TMZ. These preliminary data support the use of complementary therapeutics to augment the anti-proliferative effect of TMZ on stem-like tumor cells.

Taken together, these data suggest a strong possibility of the synergistic efficacy of anti-oxidants and TMZ. Our results indicate that LA offers the most promise as a complementary supplementation, followed by Melatonin. Both of these supplements offered a significant ability to decrease proliferation at more than one concentration. Importantly, they demonstrate activity at doses that are physiologically attainable. Especially exciting is that these two anti-oxidants augmented the action of TMZ in an in vitro model of cancer stem-like cells, suggesting the possibility of anti-proliferative effects in vivo. Before concluding that supplementation with these anti-oxidants is warranted, further studies into the mechanism of action are needed with additional GBM cell lines and neural cancer stem-like cells. Future studies may explore this end through quantitative immunohistochemistry, western blot, qPCR and flow cytometry, and sphere formation. Most importantly, the in vivo activity of the anti-oxidants studied ought to be investigated in a controlled mouse orthotopic xenograft model. Taken together, this pilot study offers a promising avenue into the potential development of a novel and effective treatment for GBM.

## 5. Conclusions

Anti-oxidants may have synergistic effects with TMZ. LA offers the most promise, followed by melatonin. Effects on reactive oxygen species may be involved, although not necessarily through glutathione peroxidase pathways.

## Figures and Tables

**Table 1:** Summary of Assays with Anti-oxidant Treatments with or without 100 µM TMZ.

	MTT U87-MG	MTT MU1454	MTT scU87-MG	MTT scMU1454	ROS Activity U87-MG	ROS Activity MU1454	GP×1 Activity U87-MG	GP×1 Activity MU1454
**Vitamin D3 100 nM**	↓***	↓*	-	-	↓**	-	-	-
**Vitamin D3 10 nM**	↓***	↔	-	-	-	-	-	-
**Vitamin D3 1 nM**	↓***	↔	-	-	-	-	-	-
**Vitamin D3 100 nM + TMZ serial**	↓***	↓*	-	-	-	-	-	-
**Vitamin D3 10 nM + TMZ serial**	↓***	↔	-	-	-	-	-	-
**Vitamin D3 1 nM +TMZ serial**	↔	↔	-	-	-	-	-	-
**Vitamin D3 100 nM + TMZ co-treatment**	-	-	-	-	↔	↑*	↓*	↔
**Melatonin 1 mM**	↓****	↓****	-	-	↔	↔	↔	↔
**Melatonin 1 µM**	↔	↓***	-	-	-	-	-	-
**Melatonin 50 nM**	↔	↓***	-	-	↔	↓**	↑*	↔
**Melatonin 1 mM + TMZ serial**	↓****	↓****	↓***	-	-	-	-	-
**Melatonin 1 µM + TMZ serial**	↔	↓****	-	-	-	-	-	-
**Melatonin 50 nM + TMZ serial**	↓****	↓****	↔	↓****	-	-	-	-
**Melatonin 1 mM + TMZ co-treatment**	↓***	↓***	-	-	↔	↔	↔	↔
**Melatonin 50 nM + TMZ co-treatment**	-	-	-	-	↔	↔	↑**	↔
**Lipoic Acid 1mM**	↓****	↓***	-	-	-	-	-	-
**Lipoic Acid 250 µM**	↓****	↓****	-	-	↓**	↓***	↓**	↔
**Lipoic Acid 100 nM**	↓***	↓**	-	-	-	-	-	-
**Lipoic Acid 1mM + TMZ serial**	↓****	↓****	-	-	-	-	-	-
**Lipoic Acid 250 µM + TMZ serial**	↓****	↓***	-	-	-	-	-	-
**Lipoic Acid 100 nM + TMZ serial**	↓***	↓***	-	-	-	-	-	-
**Lipoic acid 250 µM + TMZ co-treatment**	↓***	↓***	-	-	↓****	↓****	↑*	↔

↓ decreased; ↔ no change; ↑ increased; * *p* < 0.05; ** *p* < 0.01; *** *p* < 0.001; **** *p* < 0.0001.
